# Pupillary behavior in relation to wavelength and age

**DOI:** 10.3389/fnhum.2014.00221

**Published:** 2014-04-22

**Authors:** Luis-Lucio Lobato-Rincón, Maria del Carmen Cabanillas-Campos, Cristina Bonnin-Arias, Eva Chamorro-Gutiérrez, Antonio Murciano-Cespedosa, Celia Sánchez-Ramos Roda

**Affiliations:** ^1^Neuro-Computing and Neuro-Robotics Research Group, Optometry and Vision Science Department, University Complutense of MadridMadrid, Spain; ^2^Department of Applied Mathematics (Biomathematics), University Complutense of MadridMadrid, Spain; ^3^Department of Optometry and Vision Science, University Complutense of MadridMadrid, Spain

**Keywords:** pupil light reflex, pupillometry, wavelength, ocular predictor, mesopic conditions, aging

## Abstract

Pupil light reflex can be used as a non-invasive ocular predictor of cephalic autonomic nervous system integrity. Spectral sensitivity of the pupil's response to light has, for some time, been an interesting issue. It has generally, however, only been investigated with the use of white light and studies with monochromatic wavelengths are scarce. This study investigates the effects of wavelength and age within three parameters of the pupil light reflex (amplitude of response, latency, and velocity of constriction) in a large sample of younger and older adults (*N* = 97), in mesopic conditions. Subjects were exposed to a single light stimulus at four different wavelengths: white (5600°K), blue (450 nm), green (510 nm), and red (600 nm). Data was analyzed appropriately, and, when applicable, using the General Linear Model (GLM), Randomized Complete Block Design (RCBD), Student's *t*-test and/or ANCOVA. Across all subjects, pupillary response to light had the greatest amplitude and shortest latency in white and green light conditions. In regards to age, older subjects (46–78 years) showed an increased latency in white light and decreased velocity of constriction in green light compared to younger subjects (18–45 years old). This study provides data patterns on parameters of wavelength-dependent pupil reflexes to light in adults and it contributes to the large body of pupillometric research. It is hoped that this study will add to the overall evaluation of cephalic autonomic nervous system integrity.

## Introduction

Human ageing affects autonomic nervous system (ANS) by increasing chronic sympathetic (Seals and Esler, 2000; Hotta and Uchida, 2010) and decreasing parasympathetic nervous system activities (Kim et al., 2006; Arnold et al., 2013) in many parts of the body. This change has important implications for the maintenance of physiological function and homeostasis, and implies a risk for middle-aged and older adults developing metabolic diseases (Seals and Dinenno, 2004). Through sympathetic and parasympathetic pathways, cephalic autonomic nervous system (C-ANS) nerves control pupil size and accommodation and regulate ocular blood flow, aqueous humor production, and intraocular pressure (Neuhuber and Schrodl, 2011), all of which can affect visual function. An imbalance between them, therefore, may result in impairment in activity in some of the most relevant parts of the eye, such as the iris (Bitsios et al., 1996; Mukherjee and Vernino, 2007).

Pupil light reflex is a well-known neurological process (Barbur, 2004) influenced by age (Winn et al., 1994; Fotiou et al., 2007a). It is well established that fast increases in the light flux on the retina causes a brisk and transient constriction of the pupil. It behaves, therefore, as a servomechanism as the two C-ANS pathways (parasympathetic and sympathetic) act in a complementary fashion. Thus, an imbalance between them can result in a pupillary light reflex defect which, in turn, may be used as an indicator of certain diseases (Fotiou et al., 2007b; Jain et al., 2011) and drug consumption (Monticelli et al., 2009; Lobato-Rincón et al., 2013). Additionally, pupil pathways are wired up to respond specifically to different features of a visual stimulus, such as coherent motion (Sahraie and Barbur, 1997), color (Young et al., 1993; Tsujimura et al., 2006), variations in the levels of contrast, as well as frequency and luminance of a light stimulus (Link et al., 2006; Carle et al., 2013).

Whilst these factors have been established for some time, spectral sensitivity of the pupil reflex response has increasingly become of interest (Adrian, 2003; Vienot et al., 2010), due, basically, to discovery of melanopsin-expressing intrinsically photosensitive ganglion cells (ipRGCs), which contributes significantly to light-evoked pupillary responses (Vienot et al., 2012).

In fact, certain studies have evaluated the influence of some monochromatic lights (blue and red) in relation to age (Daneault et al., 2012), showing smaller absolute pupil areas in the older subjects that reflected a loss of autonomic control in the older individuals. On the other hand, pupillometric research using different wavelengths on visually impaired subjects with certain neurodegenerative diseases has provided useful information to distinguish outer retinal disease from healthy retinas (Leon et al., 2012) and to establish stages of severity in some disorders such as optic neuritis and multiple sclerosis (Moro et al., 2007).

It is commonly accepted that studies with larger subject samples gain a better understanding of age-related interactions according to wavelength (Daneault et al., 2012) and so this study has used 97 volunteers. As far as we know, there are no previous reports on the pupil's light reflex response to four different light-wavelengths (white light-5600°K, 450, 510, and 600 nm) in mesopic conditions (0.05–5 cd/m^2^) in a large sample population, as is the case in this study.

This study intends to gain a better understanding of the pupil's response to different light-wavelengths in regards to age to contribute to the large body of pupillometric research. Therefore, the aim of the study is to analyze the pupil's behavior and show the functional integrity of the C-ANS depending on age and the wavelength of light. Given this, our subsequent hypothesis is that pupil behavior is different between age groups for different wavelengths in mesopic conditions. Potential differences in pupillary behavior amongst ages could, therefore, provide additional diagnostic information correlating to the status of C-ANS function from either physiological or pathological origin as phasic and, specifically, tonic changes in pupillary light reflex are a general indicator reflecting autonomous arousal.

## Methods

### Participants

The study was conducted with strict adherence to the Declaration of Helsinki and the study protocol was approved by the Research Ethics Committee of the Getafe University Hospital (Madrid, Spain). Before giving consent to participate, subjects were informed about the study protocol.

Ninety seven caucasian volunteers (40 men; 57 women), aged 16–78 years, of good ocular and general health were examined. The sample was divided into two groups according to their age: younger adults [*N* = 71 (28 men; 43 women); age range: 16–45 years; mean: 26.10 ± 7.16 years]; older adults [*N* = 26 (11 men; 15 women); age range: 46–78 years; mean: 58.34 ± 9.12 years]. The cut-off age for the groups was based on potential increase of lens changes due to oxidative stress as reported in the literature (Kaur et al., 2012; Petrash, 2013; Prokofyeva et al., 2013).

Study exclusion criteria included any ocular pathology that could have potentially affected pupil light reflex response, and individuals affected by dyschromatopsias.

### Materials

Pupil diameter was monitored with the dynamic binocular Pupilometer Power Refractor II (Plusoptix, Germany). Vertical and horizontal pupil diameters (mm) were measured using the PR's edge-detection algorithm, averaged to give overall pupil diameter (Schaeffel et al., 1993). The maximum precision obtained with this pupilometer ranges from 4–8 mm (precision = 0.1 mm; error ± 0.3 mm).

**Figure 1:**
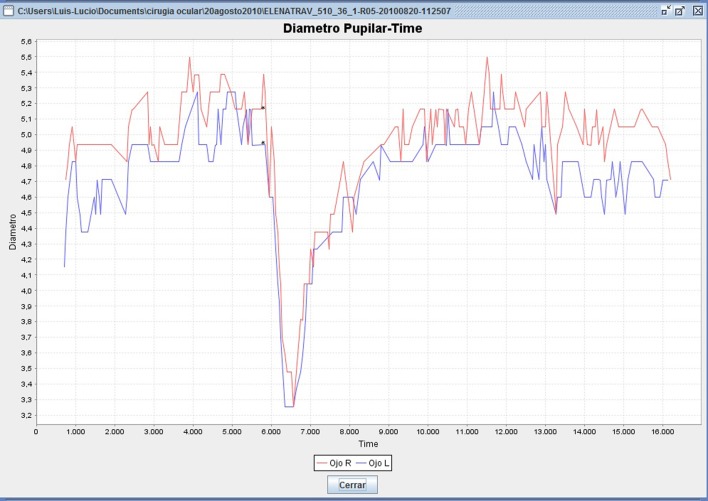
**Pupillometry menu generated by Cabanillas software (data from a 36-year-old woman).** The upper curve (red) shows OS pupil reaction; lower curve (blue) shows OD pupil reaction. Asterisks on the curve represent the instant in which the light is emitted. The Y axis represents pupil diameter (mm) and the X axis represents time of recording (ms).

Data was obtained every 0.04 s (25 measurements per second), and analyzed using a specially-designed software application (*Cabanillas software*), developed in java swing code (Sun Microsystems, USA), that parses the data and calculates the required variables into compiled CSV files (Lobato-Rincón et al., 2013). The algorithmic method of the datapath uses backtracking, which allows optimum data finding and non-matching data rejection, such as invalid measurements and noise. Figure 1 shows an example graphic of the pupil curve generated by the Cabanillas software.

Variables selected in the Cabanillas' program were: (a) Basal pupil diameter (mean pupil size from second 0 until immediately before stimulus emission, mm); (b) Latency (time before constant reaction to light stimulus, ms); (c) Amplitude of response (difference before light emission and maximum constriction, mm); and, (d) Velocity of constriction (maximum speed during pupil constriction).

The light source used for the study was the Mecablitz 60 CT-1 (Metz, Germany) flash, with a color temperature of 5600°K, completely covered by an opaque surface, except for a central 3 mm^2^ area, to obtain point light source, with an intensity measured at the cornea of 5 lux. In agreement with Bitsios et al. (1996), the duration of each light stimulus was 200 ms, as it is shorter than the average latency of the pupillary light-reflex.

The light source was connected to the PC's parallel port by an assembler with a PIC16F630/676 microcontroller (Microchip Technology, USA), thus enabling registration of an event as the instant in which the light is emitted.

Blue (450 nm), green (510 nm), and red (600 nm) monochromatic lights were obtained by the use of narrow frame-mounted interference band-pass filters (Edmund Optics, New Jersey, USA). These wavelengths are representative of the three bands in which visible spectrum is divided (short = blue, medium = green; long = red) and reproduce the effect of the photons' energy, tissue penetration and biological effects (Wu et al., 2006). Intensity of the white light was attenuated by the use of a neutral density frame-mounted filter (Edmund Optics, New Jersey, USA), thus ensuring that all wavelength-emitted intensities were the same.

Spectra through different filters were assessed with Spectrometer USB2000 (Ocean Optics, Florida, USA) and set to peak at 450, 510, and 600 nm. Irradiance levels were verified using a calibrated radiometer PM100D (Thorlabs Inc., New Jersey, USA).

### Experimental protocol

Measurements were always done between 10 am and 4 pm, in a dimly lit room, with a 0.3 cd/m^2^ background illumination, at the Neuro-Computing and Neuro-Robotics Research Group Laboratory (Faculty of Optics, Universidad Complutense de Madrid, Spain). Subjects were positioned 1 m from the pupillometer's camera.

Before recording pupillary light reflexes, subjects were adapted to the dark for 10 min in a dark room. The fixation point was established at distance-vision, thus avoiding direct near-vision possible bias source. Light stimulus was emitted after 6 s, in order to obtain stable fixation conditions. The total time for recording was 16 s. As in Daneault et al. (2012) study, exposure to different light wavelength stimuli were separated by 2 min in dark conditions. The order of the four wavelengths of light emissions was randomized.

### Statistical analysis

Generalized Linear Model (GLM) included three categorical factors: wavelength, age group, and participant code nested into the age group. Participant code was included in order to minimize inter-individual variability, thus improving statistical power.

GLM was carried out for three dependent variables: Latency, Amplitude of response, and Velocity of constriction, as these variables are parameters derived from pupil light-reflex, unlike basal pupil diameter, which was always recorded before light stimulations with the different wavelengths. For amplitude of response, participant code was substituted by the covariate baseline pupil diameter, to rule out its influence on the amplitude of the response.

GLM included interactions between wavelength and age group. Significance in these effects of interaction for the variables analyzed would mean that the differences showed in the responses according to wavelength also depended on the age group and *vice versa*. Consequently, it would allow us to analyze the influence of factors separately for each variable and perform three additional statistical analyses: Randomized Complete Block Design (RCBD) Student's *t*-test and ANCOVA (the latter was only for amplitude of response, including baseline pupil diameter as covariate).

Firstly, regarding wavelength influence, a RCBD was carried out for both age groups to highlight the fact that the responses to different wavelengths originate from the same group of individuals (paired data). In fact, RCBD can detect influences that could be unnoticed in a One-Way ANOVA. In each age-group, wavelength and participant code were used as factors. When statistical significance for wavelength was obtained, Fisher Least Significant Difference (LSD) test was used for a *post-hoc* analysis.

Lastly, to analyze the effect of age in an independent manner, data for each variable depending on the age group for each wavelength was compared by means of Student's *t*-tests. In relation to amplitude of response, however, and given that basal pupil diameter differs between the age groups, this variable was used as a covariate in an ANCOVA analysis. In addition, Student's *t*-tests were also used to determine the effect of age on the dark-adapted basal pupil diameter.

## Results

Mean mesopic basal pupil diameter across all subjects was 5.61 ± 1.1 mm (min. 3.12 mm; max. 8.47 mm).

Basal pupil diameter was always recorded before light stimulation with any of the different light wavelengths used in this study; thus, it was a non-wavelength dependant variable. As to the influence of age on this pupillary variable, an unpaired Student's *t*-test was carried out. As expected, volunteers ≤45 years showed greater basal pupil diameter (*t* = 8.17; *p* < 0.0001) than those ≥46 years (approximately 1 mm less in mesopic conditions).

### Age and wavelength effects and its interaction

Using generalized linear models, the overall analysis of the amplitude response did not reflect a significance for age but it did so for wavelength [*F*_(3, 329)_ = 10.93; *p*-value < 0.0001] and for the interaction between wavelength and age [*F*_(3, 329)_ = 4.38; *p*-value = 0.0048].

The overall analysis for velocity of constriction did not show any significance for either of the two factors (age and wavelength), nor for their interaction. Finally, the overall analysis of latency revealed significance for both factors (age [*F*_(1, 139)_ = 5.79; *p*-value = 0.0175]; wavelength [*F*_(3, 202)_ = 4.36; *p*-value = 0.0053]) and a *p*-value that was very close to the significant 0.05 [*F*_(3, 202)_ = 2.42; *p*-value = 0.06] for the interaction of the two factors. This non-statistically significant interaction could reflect certain lack of statistical power.

**Figure 2:**
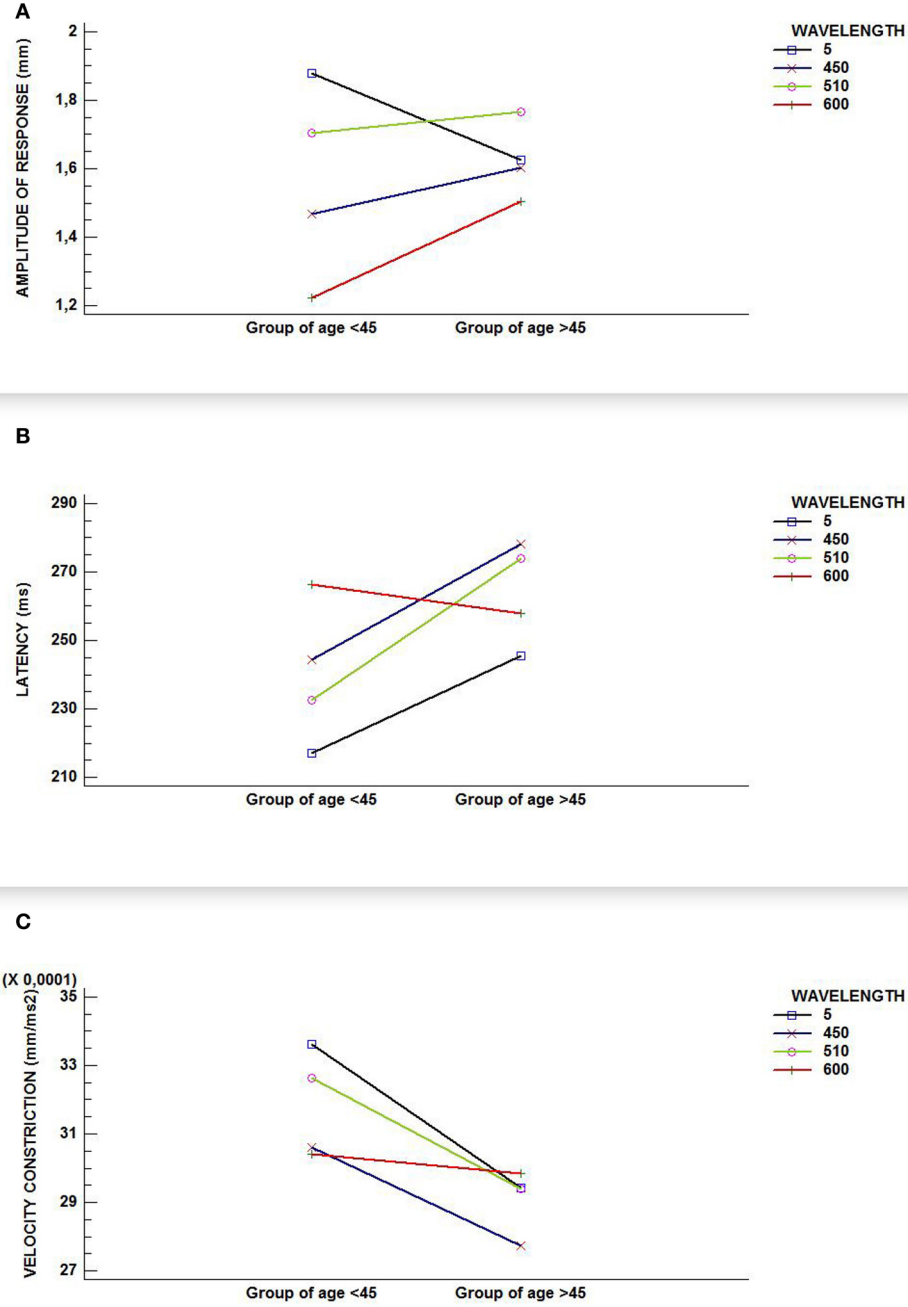
**Interaction graphics for (A) amplitude of response; (B) latency; and, (C) velocity of constriction.** Although all figures show an interaction between factors (wavelength and age group) in our sample, only **(A)** reflects a statistically significant interaction effect (*p*-value < 0.000).

As the GLM-analysis yielded statistically and near-statistically significant differences in study variables, as shown in Figures 2A,B,C (graphic interactions are seen between wavelengths and groups of age), we were encouraged to conduct RCDB-, ANCOVA-, and Student's tests-analyses for all three variables involved in the pupil light reflex. We did this in order to study the influence of the wavelength on the age groups and the influence of age on wavelength in an independent fashion, as reported above.

### Effects of wavelength in the different age groups

**Figure 3:**
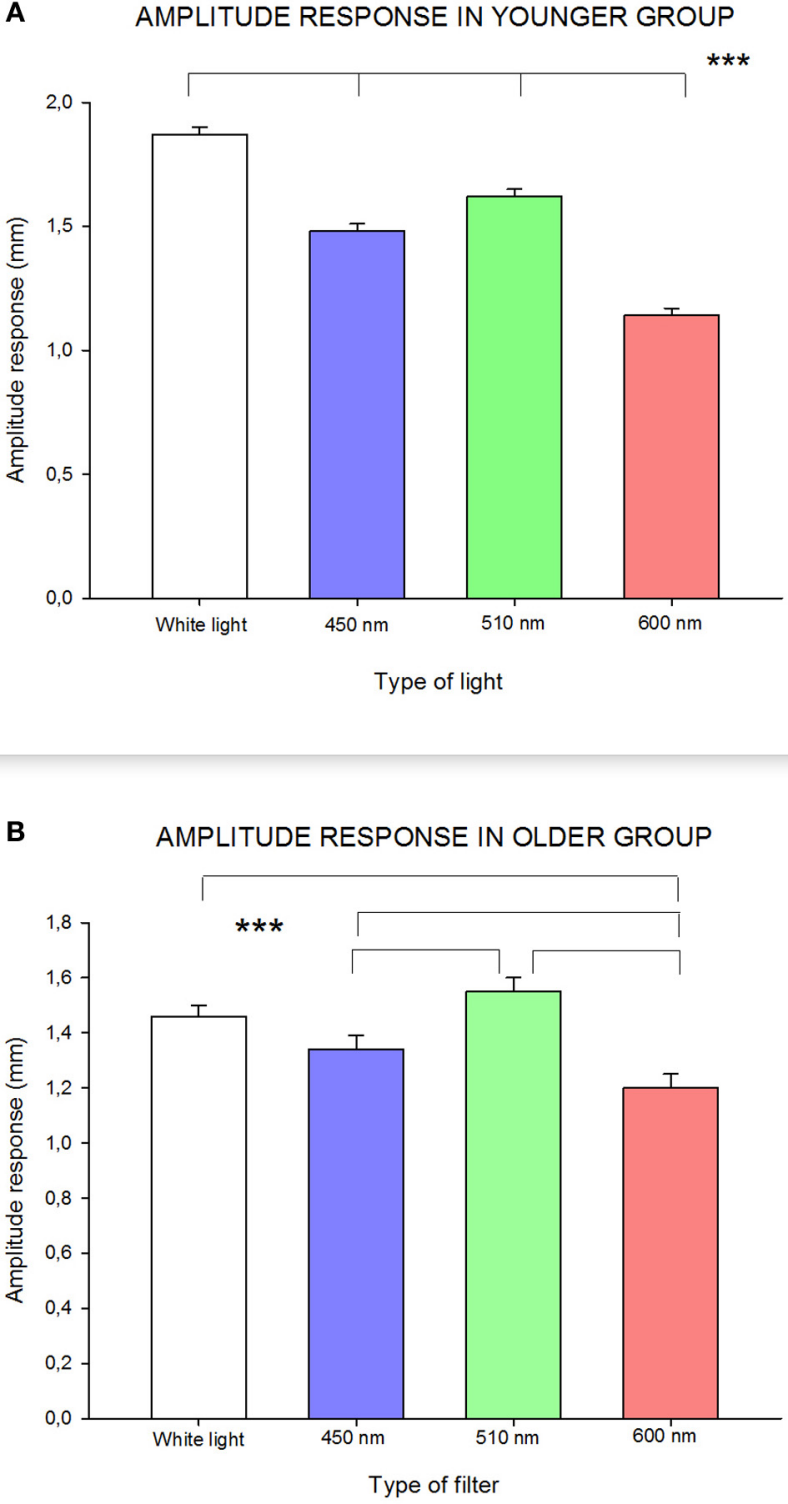
**Influence of wavelength on amplitude of response in (A) younger group; and (B) older group.** Statistically significant differences are highlighted by the three asterisks (*p*-value < 0.001) in both figures. Standard errors are shown with error bars.

As can be seen in Figure 3A, RCBD-amplitude of pupillary responses for the younger age group (≤45 years) was significantly influenced by wavelength [*F*_(3, 173)_ = 92.84; *p* < 0.0001]: white and green (510 nm) lights caused statistically significantly higher amplitude of responses, whereas red (600 nm) and blue (450 nm) wavelengths induced significantly smaller amplitude of responses. In this age group, all amplitudes of response were of statistically significant difference to each other.

In the older age group (≥46 years), pupillary response, in terms of amplitude, was slightly different than in the younger group [*F*_(3, 53)_ = 9.85; *p* < 0.0001]: there was a statistically significant lower amplitude of response to red (600 nm) compared to all other wavelengths, and with blue (450 nm) compared to green (510 nm) wavelengths (see Figure 3B).

In group ≤45 years, latency was clearly dependent on wavelength [*F*_(3, 156)_ = 9.99; *p* < 0.0001]: response to red (600 nm) was significantly longer than to any other studied wavelengths. Additionally, latency in blue (450 nm) conditions was significantly longer compared to white light. In group ≥46 years, no statistically significant differences in latency were found.

In regards to velocity of constriction by RCBD, in the younger group (≤45), statistically significant higher [*F*_(3, 173)_ = 2.90; *p*-value = 0.04] values were obtained with white and green (510 nm) compared to blue (450 nm) wavelengths. In the older age group (≥46), no statistically significant differences were found.

### Effects of the age in the different wavelength conditions

In relation to the amplitude of the pupillary response between age groups, ANCOVA analysis was carried out, with pupil basal diameter as a covariate to rule out this potentially confounding factor. Results obtained show that age had no significant effect on the amplitude of response, although in red conditions (600 nm) some effects od near significance was observed [*F*_(1, 79)_ = 3.51; *p*-value = 0.06], perhaps due to lack of power.

Student's *t*-test latency comparisons yielded significant differences statistically between the two age groups (*t* = −2.55; *p* = 0.01) in white light conditions as it was shorter in younger group compared to the older group. No statistically significant differences in latency between groups were found for any other wavelength in the study.

Velocity of pupillary constriction between groups by Student's *t*-test did not show any statistically significant differences, except for green (510 nm) wavelength. It was observed that higher results, which were statistically significant, were obtained for those ≤45 years compared to ≥46 years (*t* = 2.31; *p* = 0.02).

ANCOVA and Student *t*-test results are shown in Table 1.

**Table 1:** **Values for all pupillary parameters obtained with the four study-light divided by age**.

	**16–45 years**		**46–78 years**		***P*-value**
	**Mean ± SE**	**(Min/Max)**	**Mean ± SE**	**(Min/Max)**	
Basal pupil diameter (mm)	**5.86 ± 0.12**	**3.43/8.47**	**4.89 ± 0.20**	**3.12/6.97**	**0.000**
White light					
Latency (ms)	**215 ± 15**	**119/360**	**252 ± 26**	**120/360**	**0.01**
Amplitude of response (mm)	1.91 ± 0.1	0.56/3.14	1.44 ± 0.3	0.45/2.41	0.26
Velocity of constriction (mm/ms)	3.4 × 10^−3^ ± 0.0002	7 × 10^−4^/6 × 10^−3^	2.9 × 10^−3^ ± 0.0005	3.4 × 10^−4^/4 × 10^−3^	0.08
Blue light (450 nm)					
Latency (ms)	241 ± 15	120/321	269 ± 26	120/359	0.06
Amplitude of response (mm)	1.53 ± 0.1	0.45/2.58	1.48 ± 0.2	0.67/2.25	0.88
Velocity of constriction (mm/ms)	3.1 × 10^−3^ ± 0.0002	4.7 × 10^−4^/6.0 × 10^−3^	2.9 × 10^−3^ ± 0.0005	6.1 × 10^−4^/5.2 × 10^−3^	0.43
Green light (510 nm)					
Latency (ms)	227 ± 15	100/321	242 ± 36	120/360	0.32
Amplitude of response (mm)	1.74 ± 0.1	1.01/2.81	1.64 ± 0.2	0.34/2.47	0.22
Velocity of constriction (mm/ms)	**3.4 × 10^−3^ ± 0.0002**	**1.0 × 10^−3^/4.8 × 10^−3^**	**2.9 × 10^−3^ ± 0.0005**	**2.7 × 10^−4^/5.0 × 10^−3^**	**0.02**
Red light (600 nm)					
Latency (ms)	259 ± 15	120/360	255 ± 19	200/320	0.76
Amplitude of response (mm)	1.27 ± 0.1	0.56/2.36	1.39 ± 0.2	0.56/1.46	0.06
Velocity of constriction (mm/ms)	3.2 × 10^−3^ ± 0.0002	1.1 × 10^−3^/5.6 × 10^−3^	3.1 × 10^−3^ ± 0.0007	5 × 10^−4^/6.3 × 10^−3^	0.73

P-values are the results from Student's t-tests and ANCOVA (for amplitude of response). Data in bold highlights a statistically significant difference between the two study groups.

## Discussion

One of the most promising goals of pupillometry is to detect deficits of pupillary light-reflex as an early sign of certain severe diseases. Pupil light reflex can be used as a non-invasive, ocular predictor for C-ANS integrity (Bremner, 2009). In this study we report the influence of different light-wavelengths and age groups on relevant pupil reflex response parameters in a large sample.

Pupillometric research in large populations groups has often been carried out only with the stimulus of white light (Fotiou et al., 2007a), obtaining shorter latencies correlated to greater light reflex amplitudes (Ellis, 1981; Bergamin and Kardon, 2003). However, after the discovery of ipRGCs, pupillometric studies have shown the importance of including color stimulation (Hattar et al., 2002). It seems that at 100 cd/m^2^, these cells have peak sensitivity to 470 nm wavelength (Ishikawa et al., 2012) and they participate in the pupil light reflex response (Provencio et al., 2000; Berson, 2003; Hatori and Panda, 2010; Schmidt et al., 2011). Blue light is often used to measure ipRGC function (Herbst et al., 2012), whereas red light is used to asses outer retina functioning (rods and predominantly cones) (Leon et al., 2012).

Although some studies have evaluated chromatic stimuli to understand the mechanisms involved in the pupil's response to color (Barbur et al., 1992, 1994; Barbur, 2004; Moro et al., 2007), as far as we know there are no previous reports on large samples comparing the pupillary reflex light response to different wavelengths.

We report the pupillometric response results, in terms of the most relevant pupil light reflex parameters obtained in a sample of 97 adult volunteers (divided into two age groups), using four different wavelengths: white light-5600°K, blue (450 nm), green (510 nm), and red (600 nm), in mesopic conditions.

Across all subjects, pupillary light response had greatest amplitude and shortest latency in white and green (510 nm) light conditions. In addition, the longest wavelength (red, 600 nm) always elicited reduced amplitude responses and longer latency values than any other light stimulus, which may reflect lower sensitivity to this particular wavelength in mesopic conditions.

Various studies (Herbst et al., 2011; Leon et al., 2012) report greater constriction due to blue light compared to a photopically equivalent red wavelength. This is in line with our results, which have shown that, across both age-groups, in mesopic conditions too, blue wavelength caused greater constriction than red, even after short and decreased intensity stimulation.

Moreover, older subjects (46–78 years) showed statistically significant lower velocities of constriction to green (510 nm) and longer latency values for white light than younger subjects (18–45 years). Although amplitude of response to red (600 nm) also showed differences, these were only of small significance. These results could reflect a functional defect in the pupil light reflex with age.

In relation to age, Bitsios et al. (1996) studied transient pupil light reflex to green wavelength (565 nm). In their sample, older people showed smaller pupil size, lower amplitude of responses and decreased maximum velocity of constriction compared to younger ones. Our results are consistent in relation to velocity of constriction to green (510 nm) and in the smaller basal pupil diameters in older subjects, but not in relation to the amplitude of the response.

Our results have shown a deficit in some parameters of the pupil light reflex, with possible contradictory meanings. There is evidence that latency and amplitude of response of the pupil light reflex are due, almost exclusively, to parasympathetic activation (Heller et al., 1990; Filipe et al., 2003). Although a real deficit of parasympathetic function can not be ruled out in our case, it is also possible that our results in amplitude of response and latency reflect the prevalence of sympathetic over parasympathetic function with age (Arnold et al., 2013). In fact, sympathetic activity may act through an increased central sympathetic inhibition of the Edinger-Westphal nucleus when light stimulus is received by the system. In this way, parameters that reflect parasympathetic activity can be indirectly modulated by inhibiting of sympathetic activity.

Winn et al. (1994) determined that there was, with age, a significant reduction in pupil size at rest point over a wide range of photopic luminance levels; our findings in mesopic conditions are consistent with these authors' findings. The steady-state component of the pupillary response is induced by pupilloconstrictor Edinger-Westphal nucleus. This nucleus is adjusted by a steady-state inhibitory projection from a number of cortical areas, by decreasing the strength of the efferent signal to the sphincter muscle of the iris (Barbur, 2004), and thus keeping medium sized pupils rather than miotic pupils. We hypothesize that this inhibitory process from cortical areas can become deteriorated in older individuals, and that such lack of inhibitory input to the Edinger-Westphal nucleus explains senile miosis. Recent studies indicate a significant decline in visual cortex function in senescent macaque monkeys (Yu et al., 2006; Fu et al., 2013); this could potentially explain this lack of inhibition in humans.

Our baseline pupil diameter differences in results between age groups are in line with those reported in the literature, and show that a tonic change in the pupil diameter is a general indicator that reflects autonomous arousal, one of the activation modes of the locus coeruleus-norepinephrine function (Aston-Jones and Cohen, 2005). As the contribution of the noradrenergic deficit to cognitive dysfunction in certain neurodegenerative diseases, such as Parkinson disease, has been underappreciated (Del Tredici and Braak, 2013), it could be interesting for future studies to evaluate the pupillary light reflex as a function of wavelength in people affected by these pathologies, in order to determine possible predictors of these diseases.

In any case, the absence of precise anatomical and electrophysiological evidence prevents clear elucidation of the mechanisms of control of the pupil response with age; currently, any suggestion is mere speculation, and, single measurements may not be attributed to either parasympathetic or sympathetic modulation in all cases (Bar et al., 2009).

The present study intends to gain a better understanding of the pupil's response to different light-wavelengths as a function of age in a large sample population of healthy adults, in mesopic conditions, and provides adult wavelength dependent data patterns of most relevant pupil light reflex parameters, contributing to the large body of pupillometric research. Some relevant parameters influenced by white (5600°K), green (510 nm), and even red (600 nm) wavelengths may be used in future studies to discriminate between normal ageing and abnormal or pathological changes in the cephalic autonomic nervous functions.

We propose that future pupillometric studies of the C-ANS and ageing should include functional imaging or electroencephalography, with simultaneous recording of stimulus-evoked pupillary function parameters. This would allow us to gain a better understanding of the mechanisms involved in transient and sustained pupil responses.

### Conflict of interest statement

The authors declare that the research was conducted in the absence of any commercial or financial relationships that could be construed as a potential conflict of interest.
